# Distinct Roles for CdtA and CdtC during Intoxication by Cytolethal Distending Toxins

**DOI:** 10.1371/journal.pone.0143977

**Published:** 2015-11-30

**Authors:** Shandee D. Dixon, Melanie M. Huynh, Batcha Tamilselvam, Lindsey M. Spiegelman, Sophia B. Son, Aria Eshraghi, Steven R. Blanke, Kenneth A. Bradley

**Affiliations:** 1 Department of Microbiology, Immunology, and Molecular Genetics, University of California Los Angeles, Los Angeles, California, United States of America; 2 Department of Microbiology, Institute for Genomic Biology, University of Illinois Urbana, Urbana, Illinois, United States of America; 3 California NanoSystems Institute, University of California Los Angeles, Los Angeles, California, United States of America; University of Louisville, UNITED STATES

## Abstract

Cytolethal distending toxins (CDTs) are heterotrimeric protein exotoxins produced by a diverse array of Gram-negative pathogens. The enzymatic subunit, CdtB, possesses DNase and phosphatidylinositol 3-4-5 trisphosphate phosphatase activities that induce host cell cycle arrest, cellular distension and apoptosis. To exert cyclomodulatory and cytotoxic effects CDTs must be taken up from the host cell surface and transported intracellularly in a manner that ultimately results in localization of CdtB to the nucleus. However, the molecular details and mechanism by which CDTs bind to host cells and exploit existing uptake and transport pathways to gain access to the nucleus are poorly understood. Here, we report that CdtA and CdtC subunits of CDTs derived from *Haemophilus ducreyi* (Hd-CDT) and enteropathogenic *E*. *coli* (Ec-CDT) are independently sufficient to support intoxication by their respective CdtB subunits. CdtA supported CdtB-mediated killing of T-cells and epithelial cells that was nearly as efficient as that observed with holotoxin. In contrast, the efficiency by which CdtC supported intoxication was dependent on the source of the toxin as well as the target cell type. Further, CdtC was found to alter the subcellular trafficking of Ec-CDT as determined by sensitivity to EGA, an inhibitor of endosomal trafficking, colocalization with markers of early and late endosomes, and the kinetics of DNA damage response. Finally, host cellular cholesterol was found to influence sensitivity to intoxication mediated by Ec-CdtA, revealing a role for cholesterol or cholesterol-rich membrane domains in intoxication mediated by this subunit. In summary, data presented here support a model in which CdtA and CdtC each bind distinct receptors on host cell surfaces that direct alternate intracellular uptake and/or trafficking pathways.

## Introduction

CDTs represent an evolutionarily successful family of virulence factors encoded by more than 30 pathogenic γ- and ε-Proteobacteria [1]. Human pathogens that produce CDTs include *Haemophilus ducreyi*, a genital pathogen causing sexually transmitted chancroid, *Aggregatibacter actinomycetemcomitans*, an oral pathogen that causes localized aggressive periodontitis and multiple gastric pathogens such as enteropathogenic *E*. *coli*, *Shigella dysenteriae*, *Campylobacter* spp., and *Yersinia enterocolitica*. *Salmonella enterica* serovar Typhi, produces a related toxin referred to as typhoid toxin that recapitulates several phenotypes associated with typhoid fever including lethargy, weight loss, neutrophil depletion and death [2–4]. CDTs increase invasion, persistence and inflammation associated with infection and may also contribute to long-term pathophysiology such as infection-associated cancer [1,5–12].

Encoded in a single operon, CDTs form a heterotrimeric “AB_2_” toxin consisting of CdtA, CdtB, and CdtC subunits [13–15]. CdtA and CdtC have been proposed to function together as the two binding “B” moieties of this heterotrimeric AB_2_ toxin that deliver the active “A” moiety, CdtB, into cells [13,14]. Following binding to the host cell surface, CDTs are internalized by clathrin-dependent endocytosis and trafficked from the cell surface through the Golgi apparatus and into the endoplasmic reticulum (ER) [16,17]. CdtB is then translocated out of the ER and ultimately into the nucleus [17–19]. CdtB possesses DNase-I like activity that generates double-strand breaks in host chromosomal DNA [20,21]. In addition, CdtB was reported to have phosphatidylinositol 3-4-5 trisphosphate phosphatase activity that induces rapid apoptosis in T-cells [22]. DNase and/or phosphatase activities of CdtB cause the host cell to undergo cell cycle arrest between the G2 and M phase leading to distension and apoptosis [20,21,23–27]. Inhibiting cell cycle and/or induction of apoptosis is predicted to disrupt the normal immune and barrier functions of rapidly dividing eukaryotic cells, including lymphocytes and epithelial cells, thus providing an advantage to pathogenic bacteria [28–30].

Interaction with host cell surfaces is a critical first step required for intoxication by all bacterial toxins. However, the mechanism by which CDTs bind to host cells is not well understood and receptors for this family of toxins have yet to be definitively identified [18,31–36]. The crystal structure of Hd-CDT revealed ricin-like lectin folds in CdtA and CdtC, suggesting that carbohydrates may serve as receptors [14]. Indeed, several reports demonstrated that CDTs bind carbohydrates, though a functional role for this family of cell-surface molecules is not yet established [31,32,36,37]. Moreover, our previous studies indicate that carbohydrates are not required for intoxication by CDTs derived from various pathogens [36]. In contrast, there is strong evidence supporting a role for host-cell membrane cholesterol in toxin binding, suggesting that CDTs interact with cholesterol-rich microdomains (i.e. lipid rafts)[17,36,38–42]. Indeed, CDTs from *A*. *actinomycetemcomitans* (Aa-CDT) and *C*. *jejuni* (Cj-CDT) bind directly to cholesterol via a cholesterol recognition/interaction amino acid consensus (CRAC) motif in their respective CdtC subunits [38,41], and supplementation of Chinese hamster ovary (CHO-K1) cells with cholesterol increased sensitivity to multiple CDTs [36]. In further support of a requirement for lipid rafts, sphingomyelin synthase 1 (SGMS1), which produces the lipid-raft component sphingomyelin, is required for efficient intoxication of multiple CDTs [43]. While cholesterol and SGMS1 are critical for various CDTs, the mechanism by which lipids and/or lipid raft associated factors support intoxication has yet to be established.

The toxin-based determinants that govern host-cell binding of CDTs are also not fully defined and conflicting results exist regarding the respective contributions of CdtA and CdtC subunits to intoxication. While there is general consensus that both CdtA and CdtC contribute to host-cell binding [14,33,44–46], studies on various CDTs using a variety of target host cell types have resulted in differing conclusions regarding the sufficiency and/or necessity for these subunits. Several studies reported that both CdtA and CdtC were necessary, based on an inability of combinations of CdtA and CdtB (CdtAB) or CdtB and CdtC (CdtBC) to intoxicate host cells [13,44–48]. In addition, site-directed mutagenesis and crystal structures of CDT from *H*. *ducreyi* (Hd-CDT) and Aa-CDT defined critical roles for a patch of aromatic residues on CdtA and a hydrophobic groove formed between CdtA and CdtC [14,46,49,50]. Mutation of other regions of CdtA or CdtC also led to inactive holotoxins, further supporting roles for both subunits [14,33,37,46,49,51]. In contrast, CtdBC heterodimers derived from *A*. *actinomycetemcomitans*, *C*. *jejuni*, or *H*. *ducreyi* were reported to be sufficient for intoxication of HeLa or HEp-2 cells, albeit less efficiently than the holotoxin [33,52–56]. In these and similar studies, CdtAB dimers were not able to intoxicate HeLa, HEp-2, peripheral blood mononuclear or CHO cells [13,33,44,48,52,55,56]. However, in an apparent contradiction, Saiki and colleagues reported the CdtAB derived from *A*. *actinomycetemcomitans* intoxicated KB oral epidermal cells while CdtBC did not [57].

Given the conflicting data in the literature, the mechanisms by which CdtA and CdtC subunits support intoxication and their relative contributions to this process remain poorly defined. We reasoned that differences in reported activities of CdtAB and CdtBC heterodimers could result from differences in the source of toxin and/or target cell types [36]. Here we examined the ability of purified recombinant CdtA and/or CdtC subunits derived from *E*. *coli* (Ec-CDT) and *H*. *ducreyi* (Hd-CDT) to support intoxication of epithelial and T-cells by CdtB.

## Results

### CdtA and CdtC independently support CdtB-mediated cytotoxicity

To evaluate the relative importance of the CdtA and CdtC subunits in CdtB-mediated cellular intoxication, we first compared the dose-dependent cytotoxicity of CdtAB and CdtBC heterodimers in human T-cell (Jurkat), human epithelial (HeLa), and Chinese hamster ovary (CHO-pgsA745; “CHO-A745”) cell lines. T-cells and epithelial cells were suggested to be relevant targets for CDTs based on their rapid proliferation, which is blocked by cell-cycle induced by CdtB, and immunological and barrier functions of these cells that normally limit infection [28–30,58]. CHO cells represent a useful model for studying the role of host glycans in toxin-binding [36]. Cdt subunits were individually overexpressed in *E*. *coli*, purified and refolded together at a 1:1:1 molar ratio then repurified by size exclusion as previously reported (CDT holotoxin) [18,36], or refolded individually and mixed at a 1:1 ratio at the time of intoxication (CdtAB and CdtBC heterodimers and CdtABC heterotrimer).

**Fig 1:**
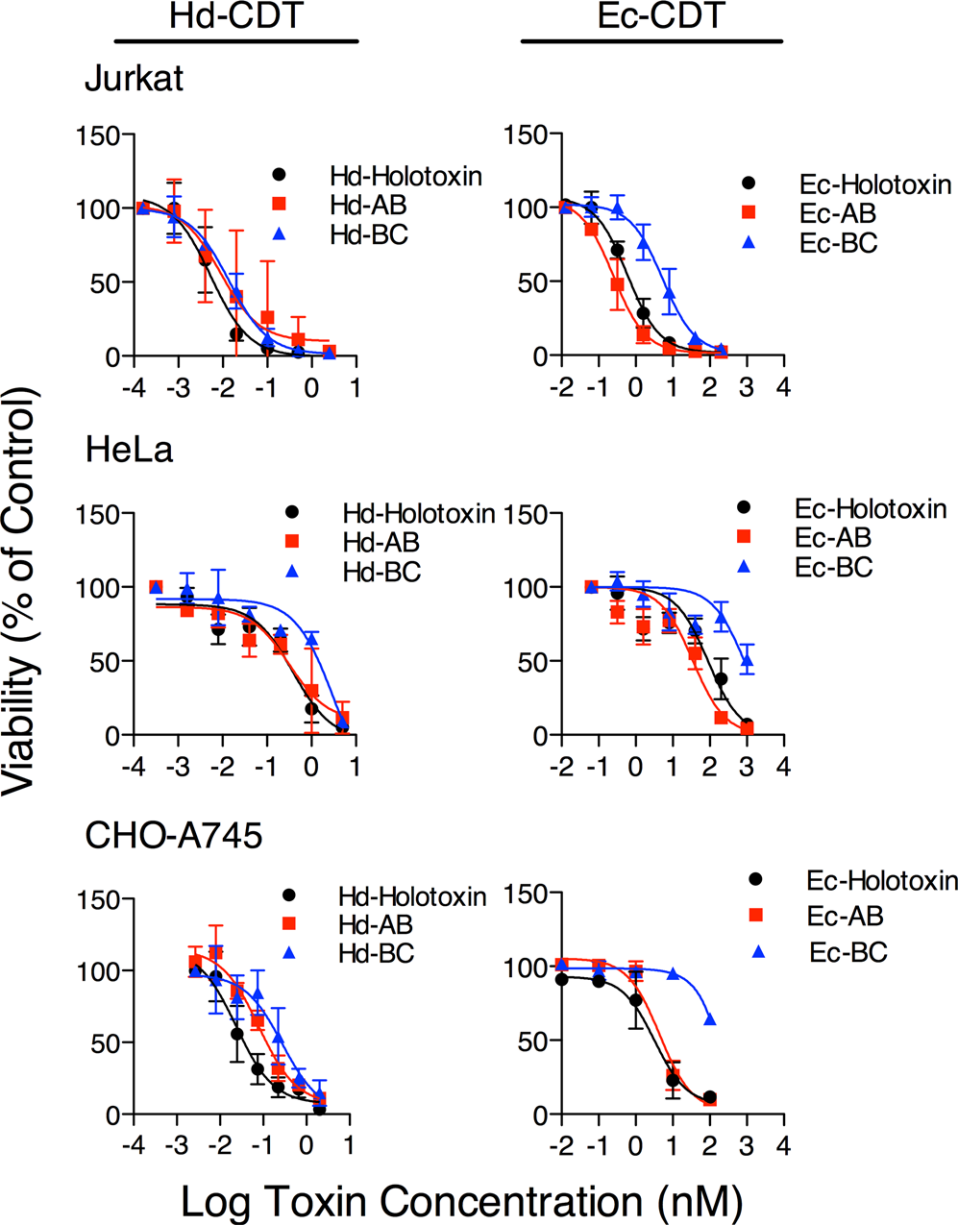
Intoxication Mediated by CdtA and CdtC Subunits. Jurkat, HeLa, or CHO-A745 cells were seeded in clear-bottom 384-well plates, incubated overnight, then challenged with the indicated toxin concentrations. Holotoxin, black circles; CdtAB, red squares; CdtBC, blue triangles. Intoxication was allowed to proceed for 48 h (Jurkat) or 72 h (HeLa and CHO-A745). Cell viability was measured by ATPlite reagent (Perkin Elmer), and normalized to ATPlite signal from unintoxicated controls. Data represent average values from three independent experiments, each performed in triplicate, +/- standard deviation. Lines represent nonlinear curve fit calculated using Prism 5 (GraphPad).

Surprisingly, the dose response curve and corresponding cellular LD_50_ values obtained for Ec-CdtAB and Hd-CdtAB heterodimers were similar to those of their respective holotoxins for all three cell lines tested (Fig 1 and Tables 1 and 2). Further, the LD_50_ value for Hd-CdtBC heterodimer was similar to that of the Hd-CDT holotoxin on Jurkat cells. In contrast, the CdtBC heterodimers derived from both *H*. *ducreyi* and *E*. *coli* were significantly less potent on HeLa and CHO-A745 cells than their cognate holotoxins (Fig 1 and Tables 1 and 2). Treatment of cells with CdtB in the absence of either CdtA or CdtC did not result in toxicity (data not shown). These data suggest that CdtA and CdtC can each independently support intoxication by the catalytic subunit CdtB, though CdtA is more efficient on the target cell types tested here while the ability of CdtC to support intoxication depends on the source of the toxin as well as on target cell. Further, these data demonstrate that CdtC is not required for cell killing by either Ec- or Hd-CDT in the three cell lines tested here.

**Table 1:** Tissue Culture LD_50_ Values for Hd-Cdt Dimers and Trimers.

Hd-CDT Cellular LD_50_ Values (pM)						
	Holo		CdtAB		CdtBC	
	LD_50_	95% CI	LD_50_	95% CI	LD_50_	95% CI
Jurkat	5.3	3.1–9.3	8.9	*2*.*0–39*	14	*9*.*7–20*
HeLa	3.8 x 10^2^	(1.7–8.5) x10^2^	3.1 x 10^2^	*(1*.*0–9*.*5) x 10* ^*2*^	32 x 10^2^	*(0*.*9–11) x 10* ^*3*^
CHO-A745	23	12–46	79	*47–1*.*3 x 10* ^*2*^	2.7 x 10^2^	*(1*.*0–7*.*2) x 10* ^*2*^
CHO-A745 + EGA	ND		ND		ND	

Average values and standard deviation (+/-) were determined from at least three biological replicates, each performed in triplicate. NT, not tested; ND, value not determined due to lack of cytotoxicity.

**Table 2:** Tissue Culture LD_50_ Values for Ec-Cdt Dimers and Trimers.

Ec-CDT Cellular LD_50_ Values (nM)								
	Holo		CdtABC		CdtAB		CdtBC	
	LD_50_	95% CI	LD_50_	95% CI	LD_50_	95% CI	LD_50_	95% CI
Jurkat	0.6	*0*.*4–0*.*8*	NT		0.3	*0*.*2–0*.*4*	5.3	*3*.*4–8*.*3*
HeLa	89	*52–1*.*5x10* ^*2*^	NT		35	*22–57*	ND	
CHO-A745	3.2	*1*.*4–7*.*7*	4.2	*2*.*3–7*.*6*	4.5	*2*.*4–8*.*3*	ND	
CHO-A745 +EGA	2.9	*1*.*4–5*.*9*	36	*11–1*.*2x10* ^*2*^	ND		ND	

Average values and standard deviation (+/-) were determined from at least three biological replicates, each performed in triplicate. NT, not tested; ND, value not determined due to lack of cytotoxicity.

### Ec-CdtC alters sensitivity to an inhibitor of endosomal trafficking.

**Fig 2:**
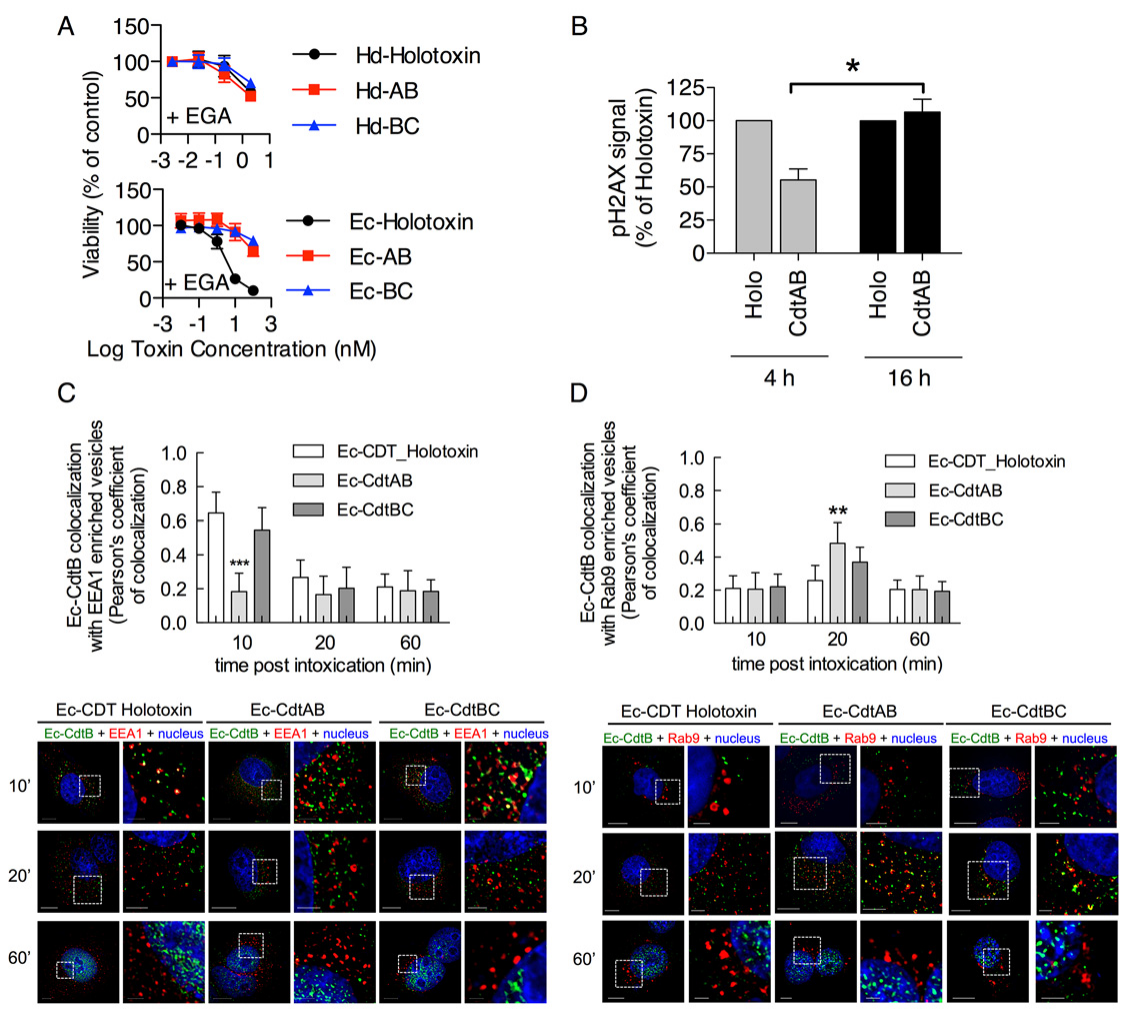
Ec-CdtC Dictates Resistance to EGA and Alters Intracellular Trafficking of Ec-CdtB. (A) CHO-A745 cells were intoxicated as in Fig 1 except that all wells were additionally treated with 12.5 μM EGA. (B) CHO-A745 cells were seeded at 8 x 10^3^ cells/well on 96-well plates and allowed to adhere overnight. The next day, cells were incubated with 1μM Ec-CDT holotoxin or 1 μM Ec-CdtAB for 4 or 16 h. Phosphorylated H_2_AX (anti-pH_2_AX) was measured by laser scanning cytometry as described in Methods. Signal intensity for pH_2_AX induced by Ec-CDT holotoxin was set at 100% and used to normalize signal from CdtAB for each time point. Graphs represent average values from three independent experiments, each performed at least 3 times. *p value = 0.0121 calculated by unpaired two-tailed t test (Prism 5, GraphPad). (C, D) CHO-A745 cells were seeded at 2 x 10^4^ cells/well on 8-well chambered slides and allowed to adhere overnight. The next day, cells were incubated on ice with 100 μM Ec-CDT holotoxin, Ec-CdtAB or Ec-CdtBC for 30 min, washed and incubated at 37°C for 60 minutes. Cells were then fixed, stained, and imaged as described in Methods [anti-Ec-CdtB (green) and EEA1 or Rab9 antibody (red)]. White scale bars at the left panel of each treatment indicate 10 μm and the right insert panel indicate 2 μm. Quantification of microscopy results was performed using Pearson's coefficient values indicating colocalization of the Ec-CdtB signal with the EEA1 or Rab9 enriched vesicles. Images and quantitation are representative of those collected from a total of 30 randomly chosen cells analyzed during three independent experiments and error bars represent standard deviations.

Although CdtC is not required for CDT-mediated cytotoxicity, this does not preclude a role for CdtC during binding or entry of the CDT holotoxin. Indeed, Damek-Poprawa and colleagues reported that CdtC from *Aggregatibacter actinomycetemcomintans* colocalizes with CdtB in the endolysosomal network and ER, and thus may influence intracellular trafficking [40]. CDTs from *E*. *coli* and *H*. *ducreyi* take distinct pathways in CHO-A745 and HeLa cells to traffic from the plasma membrane to the lumen of the ER [18,59]. Specifically, intoxication by Hd-CDT is inhibited by lysosomotropic agents that neutralize late endosome pH and by dominant negative Rab7, indicating that this toxin traffics through a late endosome prior to accessing the Golgi and ER [17,18]. Indeed, Hd-CdtB co-localizes with the late endosomal marker Rab9 [18]. In contrast, intoxication by Ec-CDT is unaltered in the presence of these inhibitors and does not colocalize with Rab9, indicating a direct early endosome to Golgi trafficking pathway [18]. These distinct trafficking pathways can be readily assessed using the small molecule EGA that prevents transport from early to late endosomes [59]. EGA blocks intoxication by Hd-CDT holotoxin, but does not block intoxication by Ec-CDT holotoxin [59](Fig 2A and Tables 1 and 2). To explore potential roles of CdtC in trafficking, we next examined sensitivity of CdtAB and CdtBC dimers to EGA. Consistent with its effect on holotoxin, EGA blocked intoxication by both Hd-CdtAB and Hd-CdtBC (Fig 2A and Tables 1 and 2). Surprisingly, EGA provided a strong block to intoxication by Ec-CdtAB but not to Ec-CDT holotoxin (Fig 2A and Tables 1 and 2). These results reveal that sensitivity or resistance of intoxication to EGA corresponds to the absence or presence of Ec-CdtC respectively, suggesting that Ec-CdtC alters the trafficking of toxin.

To further determine the importance of Ec-CdtC for the toxin’s cellular activity we assessed the ability of Ec-CdtB to induce DNA damage at early, i.e. 4 hour, and late, i.e. 16 hour, timepoints. Ec-CdtB activity in the nucleus was determined by monitoring phosphorylation of histone H_2_AX, which occurs in response to double strand breaks induced by Ec-CdtB [36,60]. Notably, this assay revealed less H2AX activation at 4 hours in cells exposed to Ec-CdtAB than Ec-CDT holotoxin (Fig 2B). While this finding appears to contrast with result presented in Fig 1 that demonstrate no difference in intoxication by Ec-CdtAB compared with Ec-CDT holotoxin, the difference may be explained by slower nuclear entry by Ec-CdtAB compared to holotoxin that is revealed here but overcome in 3–4 day cytotoxicity assays employed in Fig 1. Indeed, pH_2_AX signals induced by holotoxin and Ec-CdtAB were equivalent by 16 hours post intoxication (Fig 2B), indicating a slower or less efficient entry process for Ec-CdtB in the absence of Ec-CdtC.

To directly test whether Ec-CdtC alters intracellular trafficking of Ec-CdtB, subcellular localization of Ec-CdtB was assessed 10, 20, or 60 minutes following addition of Ec-CDT holotoxin, Ec-CdtAB dimer, or Ec-CdtBC dimer. The CdtAB dimer failed to co-localize with the early endosome marker EEA1 at any time point analyzed, while holotoxin and Ec-CdtBC localized robustly with EEA1 at 10 min post intoxication (Fig 2C). Consistent with sensitivity to EGA, the Ec-CdtAB dimer colocalized with the late endosome marker Rab9 at 20 min post intoxication, while Ec-CDT holotoxin and Ec-CdtBC did not colocalize with Rab9 at any time point assayed (Fig 2D). In summary, these results indicate that Ec-CdtC directs intracellular trafficking of Ec-CdtB through a pathway that is marked by EEA1 but not Rab9 and is insensitive to EGA. Such trafficking may result from interactions of Ec-CdtC with an unidentified receptor or other component of the host cell membrane.

### Cholesterol dependency for efficient intoxication by Ec-CDT

Having established that Ec-CdtC influences subcellular trafficking of Ec-CdtB, we next sought to determine host factors that influence sensitivity or resistance to EGA. Cholesterol in the host cell membrane is required for binding and/or intoxication by Hd-CDT, Aa-CDT, and Cj-CDT [17,38,39,41]. CdtC subunits from *A*. *actinomycetemcomitans* and *C*. *jejuni* contain CRAC domains that were proposed to bind directly to cholesterol in the host plasma membrane [38,41]. CRAC domains are defined by the amino acid sequence L/V-X_(1–5)_-Y-X_(1–5)_-R/K where ‘X’ is any amino acid. Interestingly, Ec-CdtC does not contain a CRAC domain and is completely devoid of tyrosine residues (data not shown), which are critical for CRAC-mediated binding to cholesterol [38].

**Fig 3:**
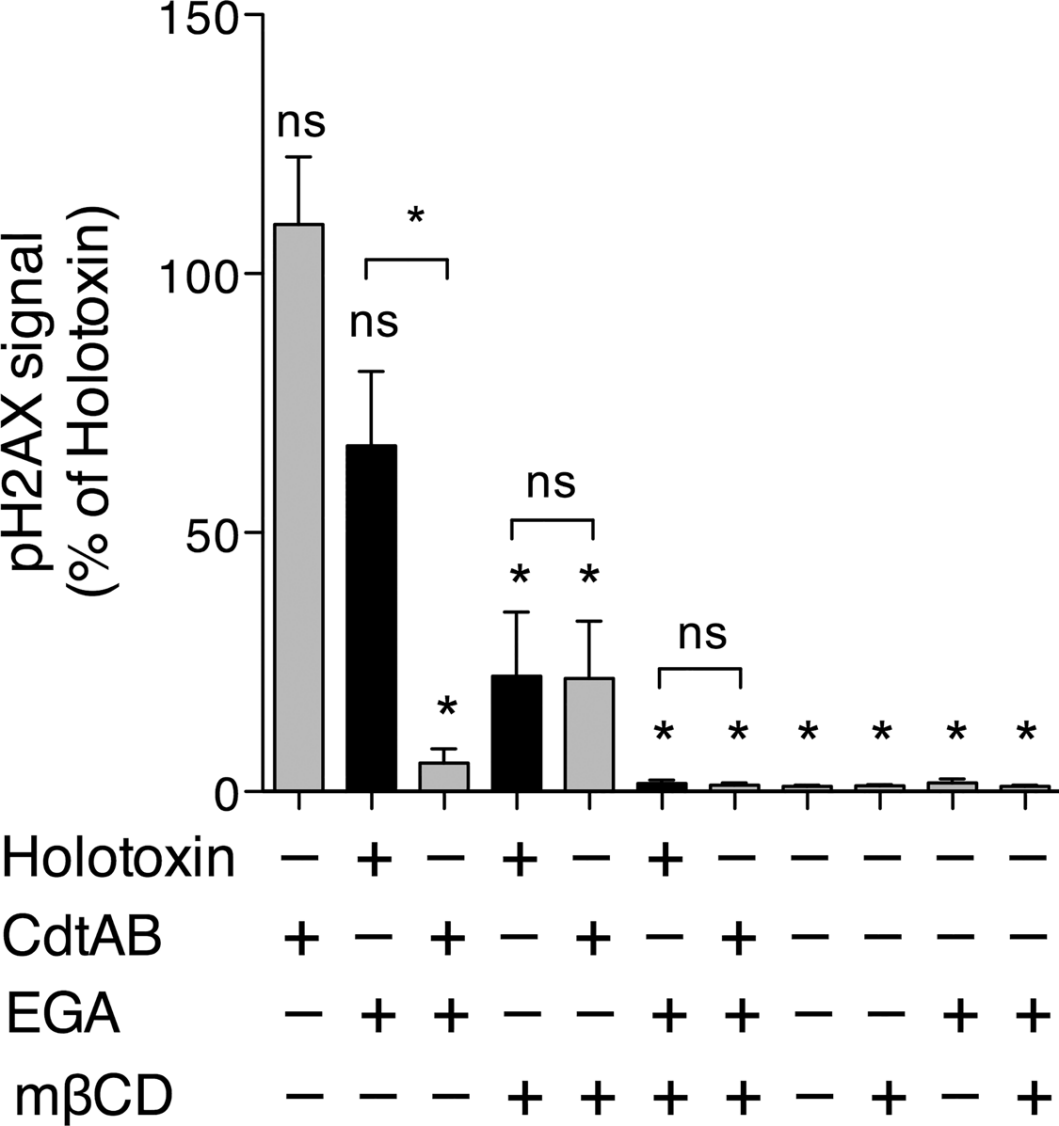
CdtC Mediates Cholesterol Dependency of Ec-CDT. CHO-A745 cells were seeded at 8 x 10^3^ cells/well on 96-well plates and allowed to adhere overnight. The next day, cells were incubated with or without 5 mM MβCD and/or 12.5 μM EGA for 1 h then challenged with 1 μM Ec-CDT or Ec-CdtAB for 16 h. Intoxication was assessed by measuring pH_2_AX by laser scanning cytometry as in Fig 2B. Data were normalized against pH_2_AX signal induced by Ec-CDT holotoxin (maximum signal) in each experiment. Graphs represent average values and SEM from three independent experiments, each performed in triplicate. All statistical analyses are from the pairwise post-test (Tukey’s) derived from one-way ANOVA. (Prism 5, GraphPad). Symbols above each column reflect comparison to Ec-CDT holotoxin (ns = not significant; * p < 0.001). Additional pairwise comparisons are indicated by brackets.

To determine whether host cellular cholesterol influences intoxication by Ec-CDT, we challenged CHO-A745 cells with holotoxin or CdtAB in the presence or absence of the cholesterol-extracting agent methyl-β-cylcodextrin (mβCD). Intoxication was determined by measuring pH_2_AX in order to avoid complications associated with reduced cell viability induced by continued presence of mβCD over the longer time course required for cytotoxicity assays (not shown). Cholesterol extraction resulted in a loss of intoxication by Ec-CDT holotoxin (Fig 3), consistent with previous reports for Hd-CDT, Aa-CDT, and Cj-CDT [17,38,39,41]. Interestingly, intoxication by Ec-CdtAB was similarly sensitive to mβCD, suggesting that Ec-CdtA dictates, at least in part, a role for cholesterol in intoxication by Ec-CDT.

We next tested whether extraction of cholesterol would sensitize Ec-CDT holotoxin to EGA. In the absence of Ec-CdtC, EGA is able to block intoxication (Figs 2A and 3), implying that Ec-CdtAB traffics through a late endosome to ultimately reach the ER lumen. We reasoned that if Ec-CdtC interacts with cholesterol or a component of lipid rafts to direct toxin trafficking into an EGA-insensitive pathway, depletion of cholesterol may likewise sensitize holotoxin to EGA. Treatment of CHO cells with both mβCD and EGA resulted in a complete block to intoxication, though the average mean pH_2_AX signal was not significantly different than that observed with mβCD alone (p>0.05 by ANOVA). Consistent with cytotoxicity assays (Fig 2A), EGA did not influence intoxication by the Ec-CDT holotoxin in cholesterol replete cells, but did block pH_2_AX signal induced by Ec-CdtAB (Fig 3). In total, these results suggest that Ec-CdtC may interact with cholesterol or a component of cholesterol-rich microdomains to alter intracellular trafficking of CdtB.

### EGA inhibits Ec-CDT mediated cytotoxicity as a function of toxin assembly.

**Fig 4:**
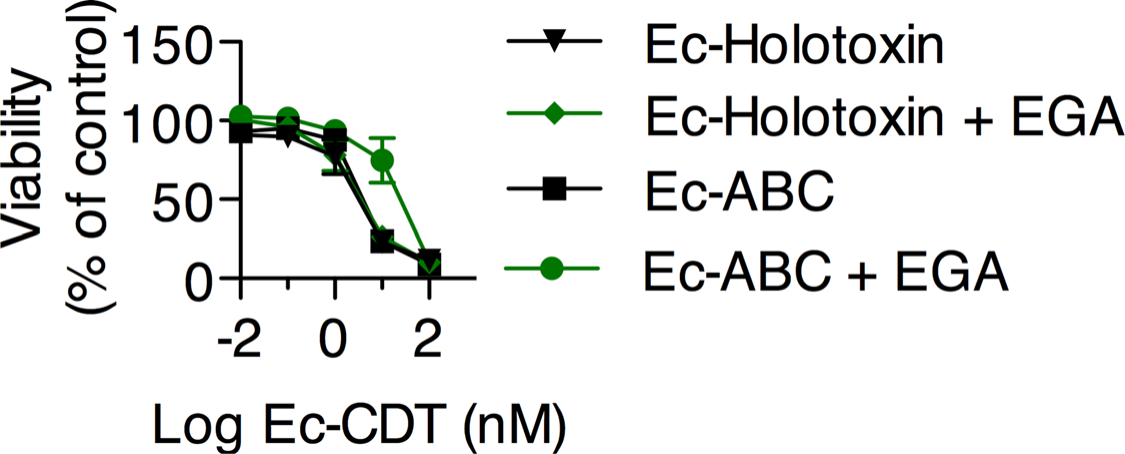
Holotoxin Assembly Method Affects Sensitivity to EGA. CHO-A745 cells were intoxicated as above in the presence or absence of 12.5 μM EGA. Additionally, cells were challenged with a combination of purified CdtA, CdtB, and CdtC subunits that were combined at the time of intoxication without further purification of assembled holotoxin (Ec-ABC). Cell viability was measured by ATPlite and normalized as above. Data represent average values from three independent experiments, each performed in triplicate.

The assembly of CDT heterotrimeric holotoxins is poorly understood. For Ec-CDT, we measured nearly identical dose response curves when CHO-A745 cells were incubated with Ec-CDT comprising purified recombinants forms of CdtA, CdtB, CdtC that were either folded together and further purified by size-exclusion (designated as Ec-holotoxin), or, refolded separately, and then mixed together just prior to addition to the cell monolayers (designated as Ec-CdtABC) (Fig 4, Table 2). As described above, the presence of EGA did not alter the dose response curves of the Ec-holotoxin (Fig 4, Table 2), consistent with the model that cellular cytotoxicity mediated by the toxin assembled from refolding all three subunits together is not dependent on cellular trafficking from early to late endolysosomal compartments. In contrast, for Ec-ABC, the presence of EGA resulted in an approximate 8-fold increase in the LD_50_ (Fig 4, Table 2). The trend towards EGA-sensitivity by toxin assembled from the mixing of the three pre-folded subunits indicates that the method of Ec-CDT toxin assembly influences the interaction of the toxin with host cells. Based on the partial block to intoxication by CdtABC, it is likely that combining toxin subunits at the time of use results in a mixed population of fully assembled holotoxin and CdtAB dimers that traffic through the host endosomal network via different routes.

## Discussion

In order to affect host cell physiology, all bacterial toxins must first interact with surface structures, i.e. receptors, present on the plasma membrane. Intracellular acting AB toxins further require endocytic uptake and trafficking to specific organelles such as late endosomes or the lumen of the ER from which they gain access to the cytosol. Two distinct subunits, CdtA and CdtC, have been proposed to support initial binding and correct sub-cellular trafficking of CDTs. However, the specific role of each subunit in intoxication is poorly understood. Here we demonstrate that CdtA and CdtC from *H*. *ducreyi* and *E*. *coli* are individually sufficient to support intoxication. CdtA from *H*. *ducreyi* and *E*. *coli* were broadly active and supported efficient intoxication of multiple target cell types as measured by cytotoxicity. In contrast, the ability of CdtC to support CdtB-mediated cell killing was more variable, with Hd-CdtC supporting intoxication more efficiently than Ec-CdtC. CdtC from both Hd- and Ec- was most functional on Jurkat T-cells. In addition, incorporation of CdtC altered trafficking of Ec-CDT in a manner that made it insensitive to EGA, an inhibitor of early-to-late endosome transport. Consistent with this model, Ec-CdtAB failed to colocalize with EEA1 but instead colocalized with Rab9 and showed reduced ability to induce pH2AX by 4 hours, indicating an altered and slower internalization and/or trafficking pathway. These data suggest that CdtA and CdtC serve distinct roles in host cell binding and trafficking and likely interact with distinct receptors on the host cell surface. Future comparative studies of the molecular content of Ec-CDT- and Ec-CdtAB-enriched vesicles (e.g. Rab protein content) will provide additional insights into the distinct trafficking pathways engaged in the presence and absence of Ec-CdtC.

Another important question is whether CDTs from different species have evolved to interact with host cells in distinct ways. Hd-CDT and Ec-CDT do not compete for specific binding to HeLa cells, indicating that they utilize different receptors for initial binding [18]. Further, Hd-CDT but not Ec-CDT displayed increased ability to intoxicate cells deficient in the UDP-galactose transporter required for incorporation of galactose into cell surface glycans [36]. Following binding, Hd-CDT and Ec-CDT also display distinct trafficking pathways to ultimately gain access to the lumen of the ER [18,19,59]. Finally, haploid cell genetic screens revealed distinct host factors required for CDTs derived from different pathogens, including Hd-CDT and Ec-CDT [43]. The differential ability of Hd-CdtBC and Ec-CdtBC dimers to intoxicate cell lines tested here may be explained by distinct receptors for CdtC from each pathogen or by altered efficiency of interactions of respective CdtC’s in interacting with the same receptor. In support of the former, the presence of Ec-CdtC correlates with resistance to EGA in CHO-A745 cells, while Hd-CdtC does not alter sensitivity to EGA (Fig 2A)[59]. So what is the host cellular component that Ec-CdtC, but not Hd-CdtC interacts with to promote resistance to EGA? Treatment of host cells with mβCD resulted in a trend towards sensitivity to EGA by Ec-CDT holotoxin, a phenotype similar to intoxication in the absence of Ec-CdtC. These results suggest that Ec-CdtC may interact with a component of cholesterol-rich microdomains to alter intracellular trafficking. On the other hand, there is evidence that cholesterol may be a common host factor required for intoxication by all CDTs [17,36,38–41]. Indeed, mβCD-mediated extraction of cellular cholesterol decreased intoxication by Ec-CdtAB as well as Ec-CDT holotoxin (Fig 3), indicating that CdtA also interacts with cholesterol or a component of cholesterol-rich microdomains to promote efficient intoxication. It is not clear whether Ec-CdtC and/or Hd-CdtC subunits interact directly with cholesterol and/or additional receptor molecules on the host cell surface. Interactions with host molecules other than cholesterol would be consistent with differential abilities of Hd-CdtC and Ec-CdtC to promote resistance to EGA. An extension of this model is that CdtC may influence the types or classes of receptor(s) used by CdtA by localizing the holotoxin to cholesterol-rich microdomains. In this case, the ability of Ec-CdtC to affect resistance to EGA would be indirect. Whether this model or an alternate, in which CdtC binds directly to a receptor that exerts a dominant effect over trafficking of the holotoxin, is correct may be answered by future identification of receptors for both subunits.

The receptors for Hd-CdtA or Ec-CdtA are unknown. Previous haploid cell genetic screening revealed that intoxication by Ec-CDT requires expression of TMEM181, an uncharacterized protein predicted to have homology to G-protein coupled receptors [43,61]. TMEM181 was reported to be expressed on the cell surface [62] and co-immunoprecipitated with Ec-CdtA [61]. However, we were unable to detect TMEM181 on the cell surface, and heterologous expression of multiple variants of TMEM181-GFP fusion proteins revealed intracellular distribution consistent with organellar localization (A. Eshraghi, unpublished). Interestingly, TMEM181 was identified in a genome-wide siRNA screen of host factors required for entry of HIV-1 [63]. Thus, TMEM181 may support intoxication by Ec-CDT in a manner that is distinct from serving as a receptor. To date, the only receptors identified for CDT family members are glycosylated proteins including PODXL and CD45, and potentially glycolipids, that support intoxication of the CDT-like typhoid toxin derived from *Salmonella enterica* serovar Typhi [4]. However, typhoid toxin does not contain CdtA or CdtC subunits, having evolved to utilize a homologue of one of the pertussis toxin heteropentameric receptor-binding “B” subunits [3,4]. Still, these studies highlight the possibility that CdtA and CdtC may each bind multiple receptors with varying efficiencies. Indeed, although the Ec-CdtBC heterodimer displayed reduced ability to intoxicate host cells compared with holotoxin, it displayed >16-fold higher activity on Jurkat T-cells (LD_50_ = 5.3 nM) than the activity of Ec-CDT holotoxin on HeLa cells (LD_50_ = 89 nM). Therefore, the identity of the target cell and by extension the presence or absence of distinct receptors, contributes along with the presence or absence of CdtA to the outcome of intoxication. Future studies to identify receptors for Cdt subunits should be careful to distinguish both the source of the CDT as well as the specific host cell from which the receptors are identified. Based on their exquisite sensitivity to CDTs and likely role in host-pathogen interactions, human T-cells are likely to be an appropriate cell type to study CDT receptors.

## Materials and Methods

### Cell culture

Proteoglycan deficient Chinese hamster ovary cells (CHO-pgsA745) were provided by Jeff Esko (UCSD) and maintained in F-12 media (Gibco) supplemented with 10% fetal bovine serum (FBS)(Atlanta Biologicals), and 100 U/mL penicillin, 100 μg/mL streptomycin, 5 mM L-glutamine (PSG)(Invitrogen). HeLa and Jurkat cells (ATCC) were maintained in Dulbecco’s Modified Eagle Medium (DMEM; Cellgro) supplemented with 10% FBS and PSG. All cells were cultured at 37°C in a humid atmosphere containing 5% CO_2_.

### Intoxication assays

Analysis of intoxication was performed either by using ATPlite reagent (Perkin Elmer) according to manufacturer recommendations or by using a modification of our previously described quantitation of pH2AX immunofluorescence [19,36]. Recombinant CDTs were cloned, expressed, and purified as described previously [36]. Each biological replicate was performed in triplicate. Intoxication data obtained by ATPlite reagent was normalized by dividing the luminescence relative light unit (RLU) signal of each replicate by the average of the unintoxicated control cells. All intoxication results presented represent average data from three biological replicates, each performed in triplicate.

For ATPlite assays, A745 and HeLa cells were trypsinized and seeded at 10^3^ cells per well in 384-well plates in a volume of 40 μL/well. Jurkat cells were seeded at 4000 cells per well. The following day, 10 μL of toxin containing medium was added to give final toxin concentrations indicated in figures. Cells were incubated for an additional 48–72 hrs followed by addition of ATPlite 1-step reagent (Perkin Elmer). Relative luminescence was measured on a Victor 3^V^ plate reader (Perkin Elmer) and values were normalized to unintoxicated controls. LD_50_ values were calculated by log transforming toxin concentrations and curve fitting data from individual biological replicates using 3-parameter nonlinear method (Prism 5; Graphpad). Data presented in Tables 1 and 2 represent average values calculated from three biological replicates.

For pH2AX assays, A745 cells were seeded at 8 x 10^3^ cells per well in clear-bottom 96-well plates (Greiner bio-one). The following day, cells were cells were treated with 5 mM methyl-β-cyclodextrin (Sigma-Aldrich) or media for 1 hr in the presence or absence of 12.5 μM EGA (4-bromobenzaldehyde N-(2,6-dimethylphenyl)semicarbazone; Chembridge). Cells were then challenged with indicated toxin for 4 or 16 hrs, fixed, permeabilized, blocked in 6% BSA and probed with anti-phospho-H_2_AX (Cell Signaling Technology) at 1:450 dilution overnight. The following day, cells were washed and probed with AlexaFluor goat-conjugated anti-rabbit at 1:500 dilution. Nuclei were then stained with 1.5 mM propidium iodide and cells were imaged by laser scanning cytometry (Acumen Explorer). The entire well was scanned for each replicate, and the number of nuclei (PI signal) positive for pH2AX was determined.

For intracellular trafficking assays, A745 Cells were seeded (2 × 10^4^ per well) in 8-well chamber slides (Thermo Scientific). After 18 h, the slides were incubated on ice for 30 min. The cells were washed two times with ice-cold PBS, pH 7.4, and then further incubated with indicated toxin in PBS, pH 7.4, with bovine serum albumin (BSA, 3%, Sigma) for 30 min on ice. After 30 min of toxin pre-binding, the cells were washed three times with ice-cold PBS, pH 7.4 then incubated with prewarmed (37°C) complete medium. After indicated time, the cells were washed with ice-cold PBS, pH 7.4, and fixed with ice-cold 4% formaldehyde. After fixing for 15 min at room temperature, the cells were permeabilized by incubating in PBS, pH 7.4, containing 0.1% Triton X-100 for 15 min, and blocked with 5% BSA for 30 min. To monitor Ec-CdtB localization in EEA1 and Rab9 enriched vesicles, cells were incubated with rabbit polyclonal anti-Ec-CdtB antibodies (1:2000 dilution); mouse monoclonal anti-EEA1 antibody (1:1000 dilution; Abcam) and mouse monoclonal anti-Rab9 antibody (1:200 dilution; Pierce) at 4°C overnight respectively, followed by incubation with goat anti-rabbit Alexa Fluor 488 antibody (1:1000 dilution; Invitrogen) and donkey anti-mouse Alexa Fluor 555 antibody (1:1000 dilution; Invitrogen) respectively at room temperature for 2 h. The cells were further counter stained for the nucleus by incubating with DAPI (100 ng/mL, Invitrogen) for 30 min at room temperature. The slides were mounted with ProLong Gold Antifade Reagent (25 μL/well; Invitrogen) and a coverslip (Thickness 0.12 mm; Fisher Scientific). Images were collected using DIC/fluorescence microscopy and deconvoluted, as described below. For each cell, images were collected from an average of 30 z-planes, each at a thickness of 0.2 μm. EEA1 or Rab9 localization analysis was conducted by using the DeltaVision SoftWoRx 3.5.1 software suite. CdtB localization into EEA1 or Rab9 were calculated from approximately 30 cells from each group over three independent experiments.

### DIC/fluorescence microscopy

Chamber slides were analyzed using a DeltaVision RT microscope (Applied Precision; Issaquah, WA), using an Olympus Plan Apo 40x oil objective with NA 1.42 and working distance of 0.17 mm DIC images were collected using a Photometrics CoolSnap HQ camera; (Photometrics, Tucson; AZ). Images were processed using SoftWoRX Explorer Suite (version 3.5.1, Applied Precision Inc). Deconvolution was carried out using SoftWoRX constrained iterative deconvolution tool (ratio mode), and analyzed using Imaris 5.7 (Bitplane AG, Zurich, Switzerland).
